# Variables associated with 90-day readmission following craniotomy for tumor in the pediatric population

**DOI:** 10.1007/s11060-025-05021-0

**Published:** 2025-04-15

**Authors:** Emal Lesha, David G. Laird, C. Stewart Nichols, L. Erin Miller, Taylor Orr, Jordan T. Roach, Christopher Troy, Brandy Vaughn, Nir Shimony, Paul Klimo Jr

**Affiliations:** 1 Department of Neurosurgery, University of Tennessee Health Science Center, Memphis, TN USA; 2 Semmes Murphey Clinic, Memphis, TN USA; 3 College of Medicine, University of Tennessee Health Science Center, Memphis, TN USA; 4 Department of Neurosurgery, Carolinas Medical Center Atrium Health, Charlotte, NC USA; 5 St. Jude Children’s Research Hospital, Memphis, TN USA; 6 Neuroscience Institute, Le Bonheur Children’s Hospital, Memphis, TN USA

**Keywords:** Brain tumor, Readmission, Predictors, Elective, Craniotomy, Pediatric

## Abstract

**Purpose:**

Readmission is a vital component of healthcare quality and is one of the core group metrics for quality-dependent outcomes. Currently, variables predictive of readmission following elective craniotomies for intracranial tumors in the pediatric population are not known. We sought to identify such variables in our population of children and young adults.

**Methods:**

All elective craniotomies for tumor resection performed at our children’s hospital from January 1, 2010, through December 31, 2022, were included for review, excluding those patients > 21 years of age. Demographic, clinical, and procedural covariates for each elective craniotomy for tumor resection were collected. Readmission was defined as readmission for any reason and to any service following discharge from the index admission (i.e., elective craniotomy). Readmission events were characterized as occurring within 90 days from discharge.

**Results:**

A total of 1,276 patients underwent a total of 1,497 elective craniotomies for tumor resection. The median age of the population at their index operations was 9.45 years, of which 58.5% of patients were male, 68.5% Caucasian, and 76.5% had private insurance. Most tumor resections were supratentorial (63.4%). There were 208 (13.9%) readmissions within 90 days of index operation, with 154 (74%) of those returning within the first 30 days. Bivariate analysis identified a number of associations, but multivariate testing found four significant predictors: age 0 to < 5 years (OR 1.55, *p* = 0.02), surgical time (OR 1.002, *p* = 0.02), high tumor grade (OR 3.15, *p* = 0.03), and return to the neurosurgical OR due to postoperative event (POE) (OR 2.81, *p* = 0.005).

**Conclusion:**

Utilizing our large pediatric tumor database, we identified key drivers of readmission following elective tumor resection. These were young children (0 to < 5 years), surgical time, high tumor grade, and return to the neurosurgical OR due to POE, of which high tumor grade was the strongest. Future studies are warranted to explore the specific ways that these predictors increase readmission risk.

**Supplementary Information:**

The online version contains supplementary material available at 10.1007/s11060-025-05021-0.

## Introduction

Since the passage of the Patient Protection and Affordable Care Act in 2010, specifically the Hospital Readmissions Reduction Program [1], readmission has become a key metric for hospitals. Often considered a core pillar for quality of health care delivery, hospitals with high readmission rates may incur financial penalties [2]. Thus, investigation into such metrics merits attention, especially in neurosurgery, where many procedures are accompanied by both high risk and cost [3, 4].

Approximately 5% and 20% of neurosurgical patients are readmitted within 7 and 30 days of discharge, respectively [2, 5]. These estimates stand out in comparison to other specialties and have been investigated in numerous studies. An analysis of patients from a single institution found an overall readmission rate of 6.9% after major neurosurgical procedures, but 14.7% after craniotomy for tumor [6]. Likewise, in comparison to non-tumor cranial procedures, 30-day readmission following craniotomy for brain tumor resection is consistently more common [3, 7, 8]. Specifically, readmission rates for patients undergoing supratentorial craniotomy for tumor have been reported as high as 17% by 30 days and 25% by 90 days [3, 6, 9].

Similar to other neurosurgical procedures, patients who undergo craniotomy for tumor removal are readmitted due to consequences from their disease or recent surgery, such as wound complications, new onset motor deficits, or seizures [10–13]. The increased susceptibility to readmission of patients with brain tumors is multifactorial. At the patient-specific level, a history of tobacco use [14] and home address within zip codes of the lowest income quartile [3] have been identified. Recent studies found that hospital craniotomy volume below the 90th percentile, Medicare coverage and non-routine discharge (i.e., discharge other than home) were associated with an increased rate of unplanned 30-day readmissions for patients with brain tumors [8].

While numerous studies have investigated readmission of adult patients undergoing craniotomy for tumor [3, 7, 9, 11], none have done so for children. Data from the pediatric National Surgical Quality Improvement Program (NSQIP-P) database identified neurosurgical procedures as the greatest predictor of readmission in pediatric patients, with surgical site infection as the most common reason [15]. Additionally, younger age has been reported as a risk factor for readmission due to surgical site infection after craniotomy for tumor resection [7]. This study aims to fill the void of data on readmission following craniotomy for brain tumors in children and young adults. We hope such analysis will have direct relevance in guiding continuing improvements in the delivery of high quality and high value health care.

### Methods

A departmental database was created and maintained by a sole research coordinator (B.V.) in which all craniotomies performed for tumor resection at the tertiary care children’s hospital were recorded prospectively for clinical research. Data analysis was completed retrospectively after the conclusion of the study period. All patients older than the age of 21 were excluded from data collection as that is the age cut-off that our hospital designates one as being an adult. All qualifying procedures from the period of Jan 1, 2010, to Dec 31, 2022, were included: elective (i.e., not urgent or emergent) craniotomy (de novo or repeat) for any degree of resection of tumor. All other cranial procedures for the treatment or diagnosis of a tumor (e.g., open biopsy, endonasal surgery, laser ablation) were excluded.

Demographic, clinical, and procedural variables were recorded (Tables 1 and 2). These included but were not limited to race, age, sex, shunt presence, primary surgeon, location and type of tumor, presence of cancer predisposition syndrome, and the occurrence of one or more medical or surgical postoperative events (POE). Age was analyzed as both a continuous and categorical variable (see additional details below): infant/toddler (0–4 years), young child (5–9 years) and pre-teen/teen/young adult (10 years and older). There were 3 surgeons (A-C) during the study period, but the vast number (94%) of the operations were performed by surgeons A and B. A patient with preexisting treated hydrocephalus was one who was dependent on a shunt or a patent third ventriculostomy at the time of their resection. Prior craniotomy was either “no” or “yes” - either at the same site or different. All tumor types were recorded, but for statistical purposes tumor grade was collapsed into either high grade or low based on histologic and molecular analysis. The few intermediate grade tumors or those that could not be graded were classified as high and low, respectively. A POE is a postoperative incident (medical or surgical, expected or unexpected) that necessitated further diagnostic testing, evaluation, or intervention. An unexpected POE can be considered synonymous with “complication”. Classifying a POE as expected versus unexpected was decided primarily by the research coordinator, taking into consideration tumor location, goal(s) of surgery, and other pertinent information. Returning to the neurosurgical OR due to POE was a variable that occurred during the index admission—after the index elective craniotomy for tumor and before discharge.

Readmission was defined as readmission to the hospital for any reason and to any service within 90 days after the date of discharge from the index admission for the elective tumor operation. If multiple craniotomies for tumor resection were conducted during a single hospital stay, then only the last craniotomy was included for analysis with the date of the last index operation used as a surrogate for hospital admission date.

### Statistical analysis

For statistical purposes, the unit of analysis is “craniotomy for tumor resection”, not the individual patient. Descriptive and frequency statistics were used to describe the demographic and clinical characteristics of the sample. Chi-square or Fisher’s Exact test were used to compare the 90-day readmission groups (yes/no) on categorical parameters in bivariate fashion. Unadjusted odds ratios (OR) with 95% confidence intervals (95% CI) were calculated for each comparison. Logistic regression analysis was performed to control for confounding variables and generate adjusted odds ratios (AOR) with 95% CI. Only categorical parameters were entered into the model and continuous parameters were dichotomized for entry into the logistic model. Statistical significance was assumed at a two-sided alpha value of 0.05 and all analyses were performed using SPSS Version 29 (Armonk, NY: IBM Corp.).

## Results

**Fig. 1:**
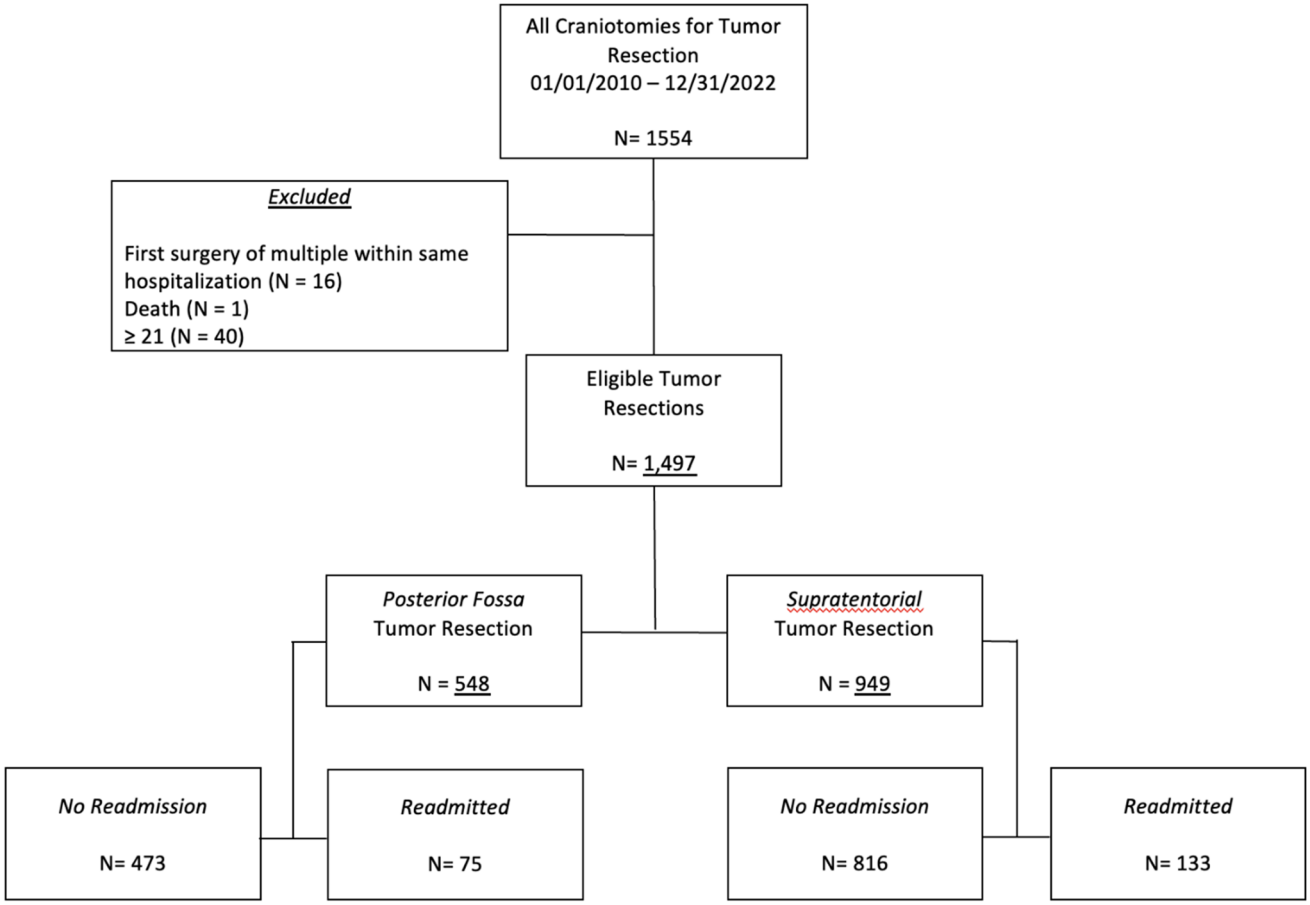
Study eligibility, inclusion criteria, and case distribution

We identified *n* = 1554 operations meeting the criteria of tumor resection craniotomies. Of those, *n* = 16 operations were excluded as the first surgery during a hospital visit requiring two (*n* = 2) elective craniotomies and *n* = 40 were above the age of 21, leaving *n* = 1498 unique encounters in our analysis. Furthermore, there was one (*n* = 1) death in this group which did not qualify for analysis, leaving *n* = 1497 total eligible encounters (Fig. 1).

Table 1 shows characteristics of the entire cohort as previously published [16]. Of the *n* = 1497 encounters, there were *n* = 1276 unique patients, *n* = 221 of which had two encounters for elective craniotomy for tumor. There were *n* = 548 (36.7%) posterior fossa tumor resections and *n* = 949 (63.3%) supratentorial. The median age at surgery was 9.45 years (IQR 4.43–14.1); the majority were male (58.5%), Caucasian (65.6%), greater than 10 years of age (40.2%), and had private insurance (76.5%). Most had a length of stay (LOS)less than or equal to seven (7) days (88.1%) and were without prior endoscopic 3rd ventriculostomy (ETV, 93.7%) or shunt (85.0%), unexpected POEs (73.9%), or a cancer predisposition syndrome (94.9%). Fifty-two (52, 3.5%) patients returned to the neurosurgical OR due to POE during the index admission.

Table 2 shows the characteristics of the patients based on readmission status and for the entire cohort. Of the *n* = 1497 encounters, *n* = 208 patients (13.9%) were readmitted within 90 days of the index operation, with *n* = 154 (74%) of them being readmitted within the first 30 days. Reasons for readmission are summarized in Online Resource 1. When compared to encounters without 90-day readmission, those readmitted within 90 days had a lower age at surgery (8.45 vs. 9.65 years), longer surgical time (287 min vs. 262 min), longer ICU stay (2.82 vs. 2.12 days), and an overall longer LOS (5.91 vs. 4.62 days) (Online Resource 2). Additionally, readmitted patients had higher rates of POEs—unexpected (34.1% vs. 24.8%), surgical (17.3% vs. 13.2%), and medical (38.0% vs. 27.5%)—when compared to patients that did not require readmission. Readmitted patients also had higher rates of reoperation by neurosurgery due to a POE during the index admission (8.7% vs. 2.6%).

**Table 1:** Encounter characteristics and patient demographics^a^

Variable	Total Encounters *N* = 1497	
Age (median)	9.45 years old	
Sex	Female	621 (41.5%)
	Male	876 (58.5%)
Race	Caucasian	982 (65.6%)
	African American	210 (14.0%)
	Hispanic/Asian/ Other	305 (20.4%)
Insurance	Private	1147 (76.5%)
	Public or none	350 (23.5%)
Tumor Location	Supratentorial	949 (63.4%)
	Posterior Fossa	548 (36.6%)
Length of stay (mean, range)	4.82 days (1–60 days)	
ICU stay (mean, range)	2.25 days (0–37 days)	
Surgical time (mean, range)	266.96 min (30–743 min)	
Return to neurosurgical OR due to POE	52 (3.5%)	
90-day readmissions (total, percent)	208 (13.8%)	

^a^Demographic and procedural information has been previously published for this patient set [16]

**Table 2:** Comparing characteristics of operations resulting in no 90-day readmission and 90-day readmission

Encounters Without vs. With 90-Day Readmissions (*N* = 1497)		No 90-day readmission (*n* = 1289)	90-day readmission (*n* = 208)
		*Number of encounters*	
Age	0 to < 5 years	331	76
	5 to 9 years	356	50
	10 + years	602	82
Race	Caucasian	845	137
	African American	174	36
	Hispanic + Other	270	35
Sex	Male	746	130
	Female	543	78
Health Insurance	Private	989	156
	Public or none	300	52
LOS	< 7 days	1147	170
	> 7 days	142	38
ICU Admission	No	153	15
	Yes	1136	193
Surgeon	A	586	105
	B	626	89
	Other	77	14
Prior ETV	No	1213	190
	Yes	76	18
Existing Shunt	No	1101	172
	Yes	188	36
Prior Craniotomy	No	661	106
	Yes, same site	509	80
	Yes, different site	119	22
Craniotomy Type	Posterior fossa tumor resection	473	75
	Supratentorial tumor resection	816	133
Tumor Grade	Low	718	91
	High	571	117
Tumor Type	Medulloblastoma	169	32
	Ependymoma	170	29
	Craniopharyngioma	94	19
	Other	153	22
	Germ cell tumors	25	2
	Low grade gliomas	457	53
	High grade gliomas	117	24
	Embryonal tumors	104	27
Medical POE	No	1118	172
	Yes	171	36
Surgical POE	No	941	130
	Yes	348	80
Unexpected POE	No	969	137
	Yes	320	71
Return to neurosurgery OR due to POE	No	1252	190
	Yes	34	18
Disposition	Home/Local housing	1254	197
	Inpatient rehab	35	11
Readmission– 30 days	No	1289	54
	Yes	0	154
Cancer Predisposition Syndrome	None	1208	198
	Known	75	9
	Unknown	6	1

The results of the bivariate and multivariate analysis are shown in Tables 3 and 4, respectively. Bivariate analysis identified a number of significant variables: Age as a continuous variable (*p* = 0.005), age category 0 to < 5 years (*p* = 0.003), LOS as a continuous variable (*p* < 0.001), LOS greater than seven (7) days (*p* = 0.003), ICU admission (*p* = 0.048), ICU stay as a continuous variable (*p* = 0.006), surgical time (*p* = 0.007), high tumor grade (*p* = 0.003), any POE (*p* = 0.015), return to the neurosurgical OR due to POE (< 0.001), and the following tumor types—embryonal type tumors (*p* = 0.002), high grade gliomas (*p* = 0.03), and medulloblastoma (*p* = 0.04). Multivariate analysis showed age category 0 to < 5 years (*p* = 0.02), surgical time (*p* = 0.02), high tumor grade (*p* = 0.03) and returning to the neurosurgical OR due to POE (*p* = 0.005) as significant predictors of 90-day readmission. Online Resource 3 further specifies readmissions among patients 0 to < 5 years.

**Table 3:** Bivariate analysis results utilized to determine association between each variable and readmission status

Variable	Bivariate			
	OR estimate	95% CI		*p*-value
Age, years	0.96	0.94	0.99	*0.005*
Age (categorical)				
0 to < 5 years	1.68	1.20	2.36	*0.003*
5–10 years	1.03	0.71	1.49	0.89
10 + years	Reference			
LOS (days)	1.05	1.02	1.07	*< 0.001*
LOS				
*≤*7 days	Reference			
>7 days	1.81	1.22	2.67	*0.003*
ICU Admission				
No	Reference			
Yes	1.73	1.00	3.01	*0.048*
ICU (days)	1.06	1.02	1.10	*0.006*
Surgical Time (minutes)	1.002	1.00	1.003	*0.007*
Race				
African American	1.28	0.85	1.91	0.23
Hispanic + Others	0.80	0.54	1.19	0.27
Caucasian	Reference			
Gender				
Female	0.82	0.61	1.12	0.21
Male	Reference			
Insurance				
Public or none	Reference			
Private	0.90	0.64	1.27	0.56
Surgeon				
A	0.99	0.54	1.81	0.96
B	0.78	0.42	1.44	0.43
Other	Reference			
Prior ETV or Shunt				
No	Reference			
Yes	1.51	0.89	2.58	0.13
Prior Craniotomy				
No + Yes, different site	Reference			
Yes, same site	0.96	0.71	1.29	0.77
Craniotomy Type				
Posterior fossa	Reference			
Supratentorial	1.03	0.76	1.39	0.86
Tumor grade				
Low	Reference			
High	1.57	1.17	2.11	*0.003*
Tumor Type				
Craniopharyngioma	1.74	0.99	3.08	0.06
Embryonal tumors	2.24	1.34	3.73	*0.002*
Ependymoma	1.47	0.91	2.39	0.12
Germ cell tumors	0.69	0.16	2.99	0.62
High grade gliomas	1.77	1.05	2.99	*0.03*
Medulloblastoma	1.63	1.02	2.62	*0.04*
Other	1.24	0.73	2.11	0.43
Low grade gliomas	Reference			
POE				
No	Reference			
Yes	1.45	1.07	1.95	*0.015*
Return to neurosurgery OR due to POE				
No	Reference			
Yes	3.49	1.93	6.30	*< 0.001*

**Table 4:** Multivariate analysis results utilized to determine association between each variable and readmission

Multivariate	OR estimate	95% CI		*p*-value
Age (years)*	N/A	N/A	N/A	N/A
Age (categorical)				
0 to < 5 years	1.55	1.07	2.24	*0.02*
5–10 years	1.03	0.70	1.52	0.89
10 + years	Reference			
LOS (days)*	N/A	N/A	N/A	N/A
LOS				
*≤*7 days	Reference			
>7 days	1.17	0.66	2.08	0.60
ICU Admission				
No	Reference			
Yes	1.32	0.73	2.42	0.36
ICU (days)	0.99	0.93	1.05	0.61
Surgical Time (minutes)	1.002	1.00	1.003	*0.02*
Tumor Grade				
Low	Reference			
High	3.15	1.10	9.05	*0.03*
Tumor Type				
Craniopharyngioma	1.93	1.05	3.54	*0.03*
Embryonal Tumor	0.57	0.17	1.89	0.36
Ependymoma	0.48	0.15	1.54	0.22
Germ Cell Tumor	0.29	0.05	1.57	0.15
High Grade Glioma	0.62	0.19	2.01	0.43
Medulloblastoma	0.64	0.20	2.08	0.46
Other	1.08	0.59	1.97	0.81
Low Grade Glioma	Reference			
POE				
No	Reference			
Yes	1.10	0.77	1.59	0.60
Return to neurosurgery OR due to POE				
No	Reference			
Yes	2.81	1.36	5.81	*0.005*

*Categorical age and LOS were chosen for multivariate analysis—see statistical methods

## Discussion

Hospital readmissions confer a significant contribution to healthcare costs, especially in neurosurgical procedures. Studies have estimated the cost of neurosurgical readmissions to be greater than $45,000 USD per stay [10], with some cases approaching $90,000 USD [9]. For perspective, examination of a state-wide California hospital database determined that a 40% reduction in the number of 30-day readmissions for brain tumor patients undergoing craniotomy would eliminate 606 hospitalizations and alleviate $12 million in cost [10].

Factors affecting hospital readmissions after craniotomies for tumor resection have been studied in the adult population, citing insurance type and patient comorbidities as major influences of 30- and 90-day readmissions [3]. The literature for the pediatric population, however, is limited. To our knowledge, this is the first publication addressing readmissions after elective craniotomy for tumor resection. Our overall 90-day readmission rate was 14%. We identified several variables on bivariate testing, but multivariate analysis resulted in only four that were statistically significant.

### Age

In our cohort, categorical age 0 to < 5 years was statistically significant for readmission compared to the other age groups. Additionally, continuous age was significant on bivariate analysis but could not be analyzed in multivariate analysis. One could posit that surgical morbidities are more likely in children of younger age. A study by Chotai et al. assessing pediatric hospital readmissions after neurosurgical procedures also found that children of younger age were more likely to be readmitted than older ones [16]. Similarly, Liu et al. found that children aged 0–3 were significantly more likely than older children to be readmitted within 30 days after craniotomy for tumor [17]. Just as plausible is that the younger the child, the more difficult it is to determine the underlying cause of any postoperative issues, leading to a lower threshold for caregivers to bring that child back to the hospital and be readmitted, whether or not a postoperative problem truly exists.

### Surgical time

We found that for each additional minute of surgical time, the chances of readmission within 90 days increased by 0.2%. The relationship between duration of craniotomy surgery and readmission risk is not well established in the literature. In a study of 9799 pediatric neurosurgical procedures, Sherrod et al. reported that each additional hour of surgery incurred a 5% increase in 30-day readmission risk on multivariate analysis [18]. Conversely, Chotai et al. found no association between surgical time and 90-day readmission among all pediatric neurosurgical procedures [16]. The more complex a craniotomy, the more time it will take which in turn may make the postoperative course longer, with higher risk of postoperative issues. Further investigation into the association between craniotomy surgery time and readmission risk is needed to better elucidate this relationship.

### Length of stay (LOS)

Like readmission, LOS is a frequently evaluated core metric, and prolonged LOS has been associated with increased likelihood of readmission in both adult [2, 3, 6] and pediatric patients [16, 17, 19, 20]. In general pediatric surgery, readmitted patients had an average LOS twice as long as patients who were not readmitted [19]. The same trend has been previously reported in neurosurgical patients, with longer LOS associated with readmission [16].

In our previous publication [21], we defined extended LOS as greater than 7 days, which is why we decided to analyze categorical LOS in the multivariate analysis and not continuous LOS. While continuous LOS and LOS > 7 days were both significant on bivariate analysis, LOS > 7 days lost its significance on multivariate analysis. Length of ICU stay also lost its significance in multivariate analysis. In the pediatric general surgery population, the relationship between the length of ICU stay and readmission events has not been clear, with their association varying between studies [16, 22]. It’s conceivable that a longer hospital stay may be reflective of a more difficult postoperative course, whether it be from symptom control (e.g., pain, nausea), complexity of the surgery, social factors or other reasons. These same reasons may persist upon discharge and be the reasons for readmission. The relationship between LOS and readmissions requires further examination.

### POEs and returning to the neurosurgical OR

While the occurrence of one or more POE (medical or surgical, planned or unplanned) did not maintain its significance on multivariate analysis, it is worth noting that readmitted patients had higher rates of POEs than non-readmitted patients. On the other hand, having to return to the OR with neurosurgery due to a POE during index admission was found to be predictive of 90-day readmission. There is limited data in the pediatric neurosurgical literature examining the relationship between multiple index operations and risk of readmission. In a study of adults undergoing any neurosurgical procedure, an increased number of operations during the index admission was predictive of neurosurgery-related readmission within 30 days [2]. It follows that patients with POEs serious enough to require additional surgical intervention prior to discharge are more likely to have a complicated post-operative course and thus are more prone to readmission.

It is possible that combining all POEs into a single group and analyzing the significance of “any POE” diminished the impact of certain POEs that are more consequential than others. In our recent publication evaluating extended LOS, POE was the strongest predictor, increasing the odds of LOS > 7 days by almost 30-fold [21]. Other studies have reported POEs as leading causesof hospital readmission after pediatric brain tumor surgery. For instance, Janjua et al., using the Nationwide Readmissions Database (NRD), found that in children undergoing intracranial tumor surgery, CNS-related complications, surgical site infections, and hydrocephalus accounted for the majority of reasons leading to readmission events [23]. Sletvold et al., assessing 30-day post-operative outcomes after pediatric intracranial tumor surgery at a single institution, found that CSF leakage and headache/nausea were the most common reasons for 30-day readmission [24].

### Tumor type and grade

We found that a high tumor grade and specific diagnoses of embryonal tumor, high-grade glioma, and medulloblastoma were predictive of future readmission events on bivariate analysis, but only high tumor grade remained significant on multivariate analysis. With respect to specific tumor types, only craniopharyngioma—compared to low grade gliomas—was a predictor of 90-day readmission on multivariate analysis, despite not being significant on bivariate analysis. While similar studies in the pediatric population are lacking, studies of adult patients have also found a diagnosis of malignant tumors to be predictive of a 30-day readmission event [8], while another adult study did not find any significant correlation between 30-day readmission and specific tumor type [11]. It is well established that craniopharyngioma resection, even if limited/subtotal, can be challenging surgically and associated with a number of potential postoperative issues. So, while it stands to reason that the average craniopharyngioma surgery is inherently more complicated than the average low grade glioma resection, we are likely only scratching the surface of what is undoubtedly a complex interconnection between tumor pathology and readmission risk.

### Strength and limitations

The major strength of this study is the quality of the data, prospectively obtained by a single individual over more than a decade. In conducting retrospective analysis of this data, it is notable that variables, such as preoperative neurologic status, precise tumor location, tumor volume, molecular classification of tumors, or goal(s) of surgery, would have added greater depth to our analysis as they could potentially impact readmission status. Regarding our definition of complication as equivalent to that of “unexpected POE”, we acknowledge that determining what is expected versus unexpected introduces an element of subjectivity and therefore potential bias. However, all data was collected by a sole research coordinator with extensive neurosurgical experience as a clinical nurse and not by the surgeon. We feel this resulted in uniform data collection while limiting surgeon bias. Additionally, as this is a single institution study, generalizability will be limited. Lastly, population characteristics are likely different to a certain degree at our institution due to the historically high volume of brain tumor patients and partnership with a quaternary-level children’s cancer hospital.

## Conclusions

This is the largest study evaluating factors (patient, tumor, surgical) associated with readmission status after elective craniotomy for tumor resection in children and young adults. Four independent covariates were identified: age 0 to < 5 years, surgical time, high tumor grade, and return to the neurosurgical OR due to POE. These findings are important for preoperative patient and family counseling, and optimization of healthcare delivery in this patient population. Future studies with a deeper analysis into the relationship between these and other potential risk factors and risk of readmission are needed.

## Electronic supplementary material

Below is the link to the electronic supplementary material.

Supplementary Material 1

## Data Availability

No datasets were generated or analysed during the current study.

## Abbreviations

LOS: Length of stay
POE: Postoperative event
