# Supplementary material for: Variables associated with 90-day readmission following craniotomy for tumor in the pediatric population
Source: J Neurooncol. 2025 Apr 15;173(3):759–67. doi: 10.1007/s11060-025-05021-0 (PMC12170774; doi:10.1007/s11060-025-05021-0)

Supplementary Information

Article Title: Variables Associated with 90-Day Readmission following Craniotomy for Tumor in the Pediatric Population

Journal name: Journal of Neuro-Oncology

Authors: Emal Lesha, MD[1-2]; David G Laird, BS[3]; C. Stewart Nichols, BS[3]; L. Erin Miller, MD[4]; Taylor Orr, MD[1-2]; Jordan T. Roach, MS[5]; Christopher Troy, MD[1-2]; Brandy Vaughn, RN[6]; Nir Shimony, MD[1,2,5,6]; Paul Klimo Jr., MD, MPH[1,2,5,6]

Affiliations:

1. Department of Neurosurgery, University of Tennessee Health Science Center, Memphis, Tennessee
2. Semmes Murphey, Memphis, Tennessee
3. College of Medicine, University of Tennessee Health Science Center, Memphis, Tennessee
3. Department of Neurosurgery, Carolinas Medical Center Atrium Health, Charlotte, NC, USA
4. St. Jude Children's Research Hospital, Memphis, TN, USA
6. Neuroscience Institute, Le Bonheur Children's Hospital, Memphis, Tennessee

Corresponding Author:
Emal Lesha, MD
UTHSC, Department of Neurosurgery
847 Monroe Ave, Suite 427
Memphis, TN 38163
Phone: (901) 448-6375
Email: elesha@uthsc.edu

Supplementary Figures Captions

Online Resource 1 Reasons for readmission
Online Resource 2 Analysis of 90-day readmission by age of patient, surgical time, ICU stay, and length of stay
Online Resource 3 Readmissions Among Patients 0 to <5 Years Categorized by (A) Relation to Craniotomy and (B) Top Five POEs

Online Resource 1

Reasons for Readmission					
**Due to POE from craniotomy**	**Total** 131	**Not due to POE from craniotomy**	**Total** 77	**Unplanned return to OR**	**Total** 94
Surgical POE	123	Planned second procedure	46	*Not due to POE*	*4*
Medical POE	4	Seizure	6	Shunt placement/ revision for HCP	3
Surgical + Medical POE	4	Fever/nausea/vomiting	7	Emergent tumor resection	1
		Obstructive HCP from tumor growth	5	*Due to POE*	*90*
		Shunt malfunction	3	Shunt revision/ replacement for HCP	39
		Head trauma	3	Wound revision	28
		Psychiatric issues	2	CSF repair leak	21
		Shunt infection	1	Sinus repair	1
		Pseudomeningocele not due to POE	1	Hickman catheter placement	1

Online Resource 2

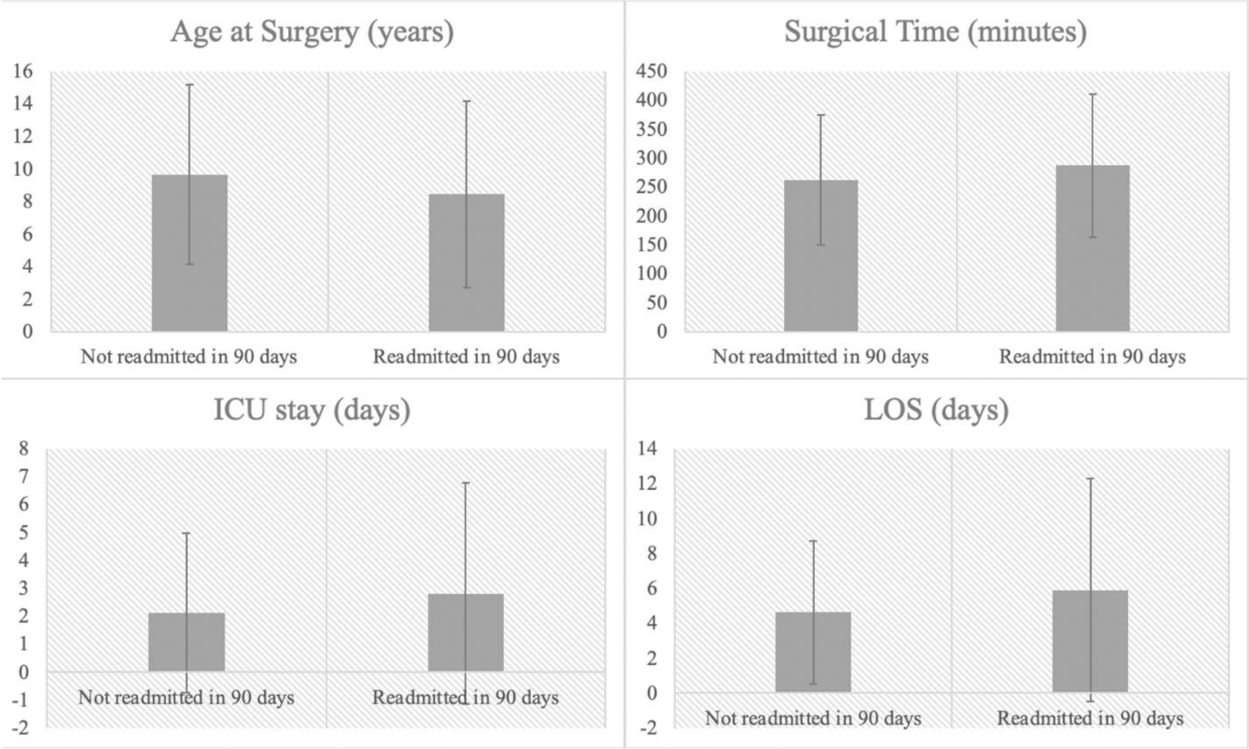

Online Resource 3

(A)

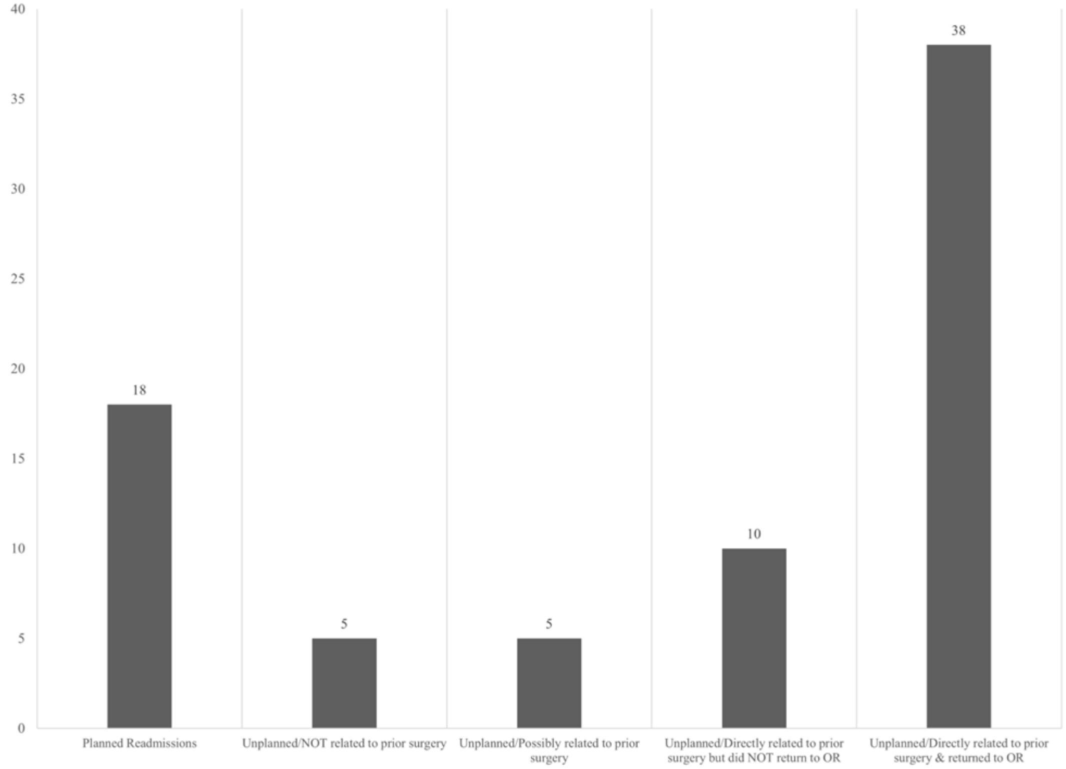

(B)

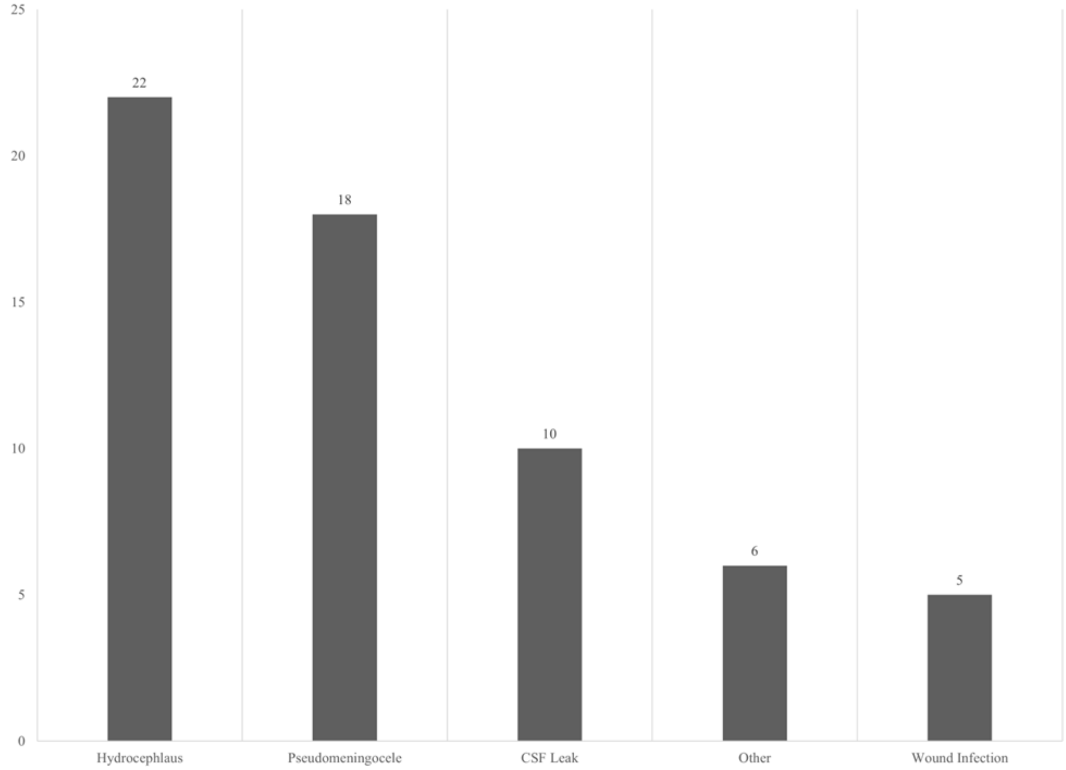

Supplement: Supplementary file 1 — Supplementary Material 1 [file 11060_2025_5021_MOESM1_ESM.pdf]
